# The Road Toward Reproducibility of Parametric Mapping of the Heart: A Technical Review

**DOI:** 10.3389/fcvm.2022.876475

**Published:** 2022-05-06

**Authors:** Augustin C. Ogier, Aurelien Bustin, Hubert Cochet, Juerg Schwitter, Ruud B. van Heeswijk

**Affiliations:** ^1^Department of Diagnostic and Interventional Radiology, Lausanne University Hospital, University of Lausanne, Lausanne, Switzerland; ^2^IHU LIRYC, Electrophysiology and Heart Modeling Institute, Université de Bordeaux, INSERM, Centre de Recherche Cardio-Thoracique de Bordeaux, U1045, Bordeaux, France; ^3^Department of Cardiovascular Imaging, Hôpital Cardiologique du Haut-Lévêque, CHU de Bordeaux, Avenue de Magellan, Pessac, France; ^4^Cardiac MR Center, Cardiology Service, Lausanne University Hospital, University of Lausanne, Lausanne, Switzerland

**Keywords:** relaxation times, cardiovascular magnetic resonance (CMR), review, reproducibility, heart, quantitative MRI (qMRI), parametric mapping

## Abstract

Parametric mapping of the heart has become an essential part of many cardiovascular magnetic resonance imaging exams, and is used for tissue characterization and diagnosis in a broad range of cardiovascular diseases. These pulse sequences are used to quantify the myocardial T_1_, T_2_, ${\text{T}}_{2}^{*}$, and T_1ρ_ relaxation times, which are unique surrogate indices of fibrosis, edema and iron deposition that can be used to monitor a disease over time or to compare patients to one another. Parametric mapping is now well-accepted in the clinical setting, but its wider dissemination is hindered by limited inter-center reproducibility and relatively long acquisition times. Recently, several new parametric mapping techniques have appeared that address both of these problems, but substantial hurdles remain for widespread clinical adoption. This review serves both as a primer for newcomers to the field of parametric mapping and as a technical update for those already well at home in it. It aims to establish what is currently needed to improve the reproducibility of parametric mapping of the heart. To this end, we first give an overview of the metrics by which a mapping technique can be assessed, such as bias and variability, as well as the basic physics behind the relaxation times themselves and what their relevance is in the prospect of myocardial tissue characterization. This is followed by a summary of routine mapping techniques and their variations. The problems in reproducibility and the sources of bias and variability of these techniques are reviewed. Subsequently, novel fast, whole-heart, and multi-parametric techniques and their merits are treated in the light of their reproducibility. This includes state of the art segmentation techniques applied to parametric maps, and how artificial intelligence is being harnessed to solve this long-standing conundrum. We finish up by sketching an outlook on the road toward inter-center reproducibility, and what to expect in the future.

## Introduction

In recent years, cardiac magnetic resonance (CMR) parametric mapping has seen a steady increase in use in the clinical setting. Parametric mapping is the quantification of one or more of the drivers of MR contrast, the relaxation times, in each pixel of an image. These relaxation times or relaxation parameters are quantifiable properties of a tissue in a magnetic field, and strongly depend on physiological properties of that tissue. This leads to large advantages of parametric maps over qualitative imaging, since these maps should no longer be dependent on scan-specific parameters such as radiofrequency (RF) coil proximity, receiver chain efficiency, or magnetic field inhomogeneities. Quantified relaxation times also reduce inter-observer variability, allow for a patient's tissue parameters to be tracked through therapy, and allows for individual patient values to be compared. In theory, these parameter maps should thus be highly reproducible, since they only depend on the interaction of physics and biology. Unfortunately, while parametric mapping can indeed uniquely and quantitatively inform on tissue properties such as interstitial fibrosis and iron deposits, most mapping techniques are in practice not as independent of confounding influences as described above. Different vendors and even different scanners at the same magnetic field strength in the same hospital often led to different baseline relaxation times. Because of these differences, the latest international consensus statement on parametric mapping of the heart (1) recommends establishing reference values in healthy volunteers for each mapping technique, scanner, and hospital. Similarly, recent international guidelines on CMR reference ranges report broad vendor-specific reference ranges (2). This indicates that parametric mapping is currently reproducible at the level of the individual MR scanner, but that relaxation parameters can often not be directly compared between different mapping techniques, or between different hospitals.

The goal of this technical review is therefore to give a overview of current parametric mapping and to project its road toward more general reproducibility. It is intended both as a primer for those new to the field and as an update on the state of the art for those stuck in it. To this end, we will first briefly describe several ways to evaluate the quality and reproducibility of a mapping technique, such as accuracy and sensitivity, and will follow this with basic links between the physics and biology of relaxation times in the heart. Next, we will give an overview of commonly applied techniques to map relaxation times as well as limitations in reproducibility of these techniques. This will be followed by an overview of recent technical improvements from a reproducibility angle, and we will finish with an outlook of what we should expect or try to bring about in the near future.

## General considerations

### Metrological Terminology

We will define several metrological and mathematical principles that are related to reproducibility so that we can discuss the various mapping techniques in unambiguous terms (3, 4). **Reproducibility** itself is the degree to which the result of an experiment can be repeated by a different team with a different setup. It should not be confused with **repeatability** (same team, same setup) or **replicability** (different team, same setup). Most parametric mapping techniques are highly repeatable and decently replicable. It should also be noted that a repeatable measurement should be **robust**, meaning that it should continue to be sensitive and accurate in the same center and with the same team in the presence of small variations in experimental conditions that are not always present [in CMR this often means motion (5) and magnetic field inhomogeneities (6)].

The reproducibility of a mapping technique can be described in more detail in terms of accuracy and precision: these characteristics should be determined for all mapping techniques for a given indication and relaxation time. Here, **accuracy** is defined as the closeness to a gold-standard technique (such as a spin echo (SE) pulse sequence). The term accuracy is often used in a qualitative sense, while its numerical value is indicated by the **bias**. **Precision** is how close multiple measurements are to one another and is often also used qualitatively. Its quantitative (and inverse) representation is the **variability**, which is generally measured as the standard deviation, coefficient of variation (i.e., the standard deviation divided by the mean), or interquartile range (IQR). Accuracy can be derived from the mean relaxation times of a tissue measured with two techniques, while precision is often taken as the inverse of the standard deviation of that relaxation time in a region of interest (ROI).

By setting a cut-off value for a relaxation time that indicates disease, one can assess the sensitivity and specificity of a mapping technique for that disease. Here, **sensitivity** is the true positive rate: the percentage of true disease cases we positively identify with our defined cut-off. **Specificity** is the true negative rate, or the percentage of true disease-free cases we correctly ruled out with our cut-off.

While the majority of parametric mapping studies report measures of accuracy and precision, most techniques are too early in their scientific testing cycle to have set cut-offs, and thus do not have globally accepted sensitivities and specificities.

### Myocardial Relaxation Parameters

The most common parametric mapping techniques in the heart quantify the T_1_, T_2_, ${\text{T}}_{2}^{*}$ (“T_2_-star”), and T_1_ρ (“T_1_-rho”) relaxation times. They were first described by Bloch (7), Bloembergen et al. (8), and Redfield (9) in their seminal papers, and are at times discussed as relaxation rates R_n_ = 1/T_n_. Relaxation times are characteristic times of decay curves that describe their respective relaxation, and strongly depend on the interactions of water molecules with their surroundings. A measured relaxation time in a pixel is an averaged result of several environments and processes, such as intracellular and extracellular water. While the tissue of a subject will have a relaxation time that we want to measure, even these “true” relaxation times in a voxel will thus be a representation of several compartments (and sub-compartments) with a unique relaxation time each. Furthermore, even these true values may significantly differ from a population average due the subject's age, gender, and other factors (2).

**T**_**1**_ **relaxation** is the increase of longitudinal magnetization and is mainly caused by an irreversible energy loss to the surroundings. The longitudinal relaxation of a signal S follows:


(1) $$\begin{array}{l} S\left(t\right)=S_{0}\left(1-Ae^{-\frac{t}{T_{1}}}\right), \end{array}$$

where A = 1 to describe recovery after saturation and A = 2 to describe an inversion (although it can be any value between 0 and 2), *S*_0_ is the equilibrium signal, and *t* is the weighting duration. Longitudinal relaxation of the myocardium is relatively efficient for ordered watery tissues such as the healthy myocardium at clinical field strengths, and its energy loss becomes slower (and the relaxation time is therefore longer) both when there is an increase in free water and when there is an increase in large molecules (10). The former occurs in the case of edema, and the latter in the case of interstitial fibrosis or amyloid deposition, making myocardial T_1_ relaxation sensitive to both of these processes. The T_1_ relaxation can furthermore be used to calculate the myocardial extracellular volume (ECV) fraction (11), which is highly sensitive to diffuse and chronic myocardial injuries. The ECV can be calculated by assuming an equilibrium exchange between the blood and myocardium for a gadolinium-based contrast agent (GBCA) (12), and combining the partition coefficient of the GBCA with the hematocrit (Ht). Ht is the volume percentage of red blood cells in blood, and is a robust approximation of the volume that a GBCA cannot flow into in blood. The partition coefficient P of the GBCA can in turn be established from the T_1_ relaxation times in the myocardium and blood pre- and post-injection of the GBCA:


(2) $$\begin{array}{l} ECV=P\left(1-Ht\right)=\left(1-Ht\right)\frac{\left(R_{1myo,post}-R_{1myo,pre}\right)}{\left(R_{1blood,post}-R_{1blood,pre}\right)}. \end{array}$$

To correctly measure the ECV, steady-state GBCA concentrations in the various compartments must be achieved, which depends on the GBCA dose and typically takes 10–15 min for scar tissue and standard doses. When Ht is not available, a “synthetic Ht” can be derived from its linear relation with the pre-GBCA blood T_1_ relaxation time, resulting in a synthetic ECV (13).

**T**_**2**_ **relaxation** is the decrease of transverse relaxation and is mainly caused by an energy exchange between spins that results in a dephasing of their magnetization (7), and can in practice never be slower than T_1_ relaxation in biological systems. Its exponential decay can be described by:


(3) $$\begin{array}{l} S\left(t\right)=S_{0}e^{-\frac{t}{T_{2}}} \end{array}$$

In the myocardium, T_2_ relaxation is acutely sensitive to the average degree of freedom of water (14, 15). If large proteins denature and release their bound water as free water (intracellular edema), T_2_ relaxation becomes less efficient and immediately slows down. When extracellular edema occurs, the T_2_ relaxation time increases further. Conversely, in the case of dehydration, there is less free water, and T_2_ values decrease.

${\text{T}}_{2}^{*}$ **relaxation** is the *effective* transverse relaxation and is the sum of T_2_ relaxation and the effect of macroscopic magnetic field inhomogeneities ΔB_i_ (8). Due to these inhomogeneities, the dephasing of the magnetization is faster than pure T_2_ relaxation. ${\text{T}}_{2}^{*}$ relaxation can be described as:


(4) $$\begin{array}{l} \frac{1}{T_{2}^{*}}=\frac{1}{T_{2}}+\gamma {\Delta B}_{i}, \end{array}$$

where γ is the gyromagnetic ratio. Since ${\text{T}}_{2}^{*}$ is sensitive to ΔB_i_, it is particularly sensitive to biological processes that cause magnetic field inhomogeneities. In the myocardium this mostly means the excessive storage of iron complexes, which may be caused by hemochromatosis (hereditary or post-transfusion iron overload) or intramyocardial hemorrhage (after acute myocardial infarction). Such excess iron (especially in the context of long-term transfusion therapies) is difficult for the body to remove by itself, and requires toxic chelation therapy. This therapy needs to be carefully dosed to reduce its side effects and, importantly, to increase patient compliance. Sources of iron can be methemoglobin (involved in hemorrhage), ferritin, and hemosiderin (involved in iron overload). It can be noted that T_1_ and T_2_ relaxation also decrease in the presence of concentrated iron compounds, but to a lesser degree.

Finally, **T**_**1**_**ρ** **relaxation** is T_1_ relaxation in the r(h)otating frame (9, 16). Despite its name containing the term T_1_, it is a transverse signal decrease, behaves like T_2_ relaxation in Equation 2, and has relaxation times between those of T_1_ and T_2_ relaxations. T_1_ρ relaxation can be achieved by spin-locking (SL) the magnetization in the transverse plane with a continuous low-amplitude RF pulse that prevents normal T_2_ relaxation. Under the influence of this SL pulse, the transverse magnetization can interact with its surroundings; hence the T_1_ name. However, the frequency ω_SL_ at which this interaction can take place is directly proportional to the amplitude B_SL_ of the SL pulse (since frequency ω_SL_ = γB_SL_), which for these low-power pulses means that the magnetization can only interact with molecules that slowly tumble in the low-kHz range: the domain of macromolecules. In the myocardium, this means that T_1_ρ relaxation is very specifically sensitive to increased concentrations of collagen, amyloid, and other large proteins. Contrary to the other relaxation types, T_1ρ_ relaxation depends not only on the physiology and the main magnetic field, but also on the applied spin-lock frequency.

## Routine Myocardial Parametric Mapping

**Figure 1:**
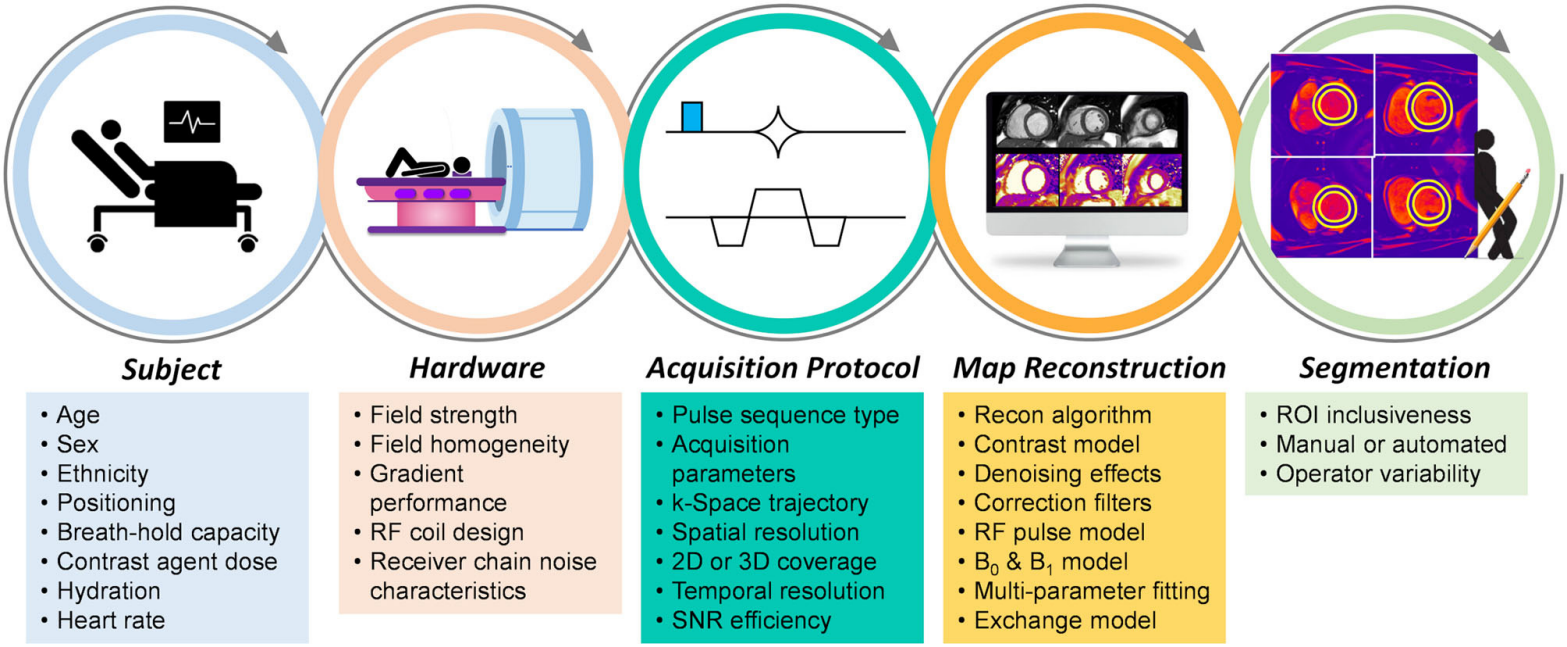
Sources of bias and variability in the myocardial mapping workflow. Various factors at each step contribute to the bias and variability of the obtained relaxation time when using myocardial parametric mapping. Not all factors contribute significantly to all mapping techniques, while some factors cancel one another, and some are included on purpose to increase sensitivity.

Here we will discuss common myocardial parameter mapping techniques that have routinely been applied in clinical practice, and compare their advantages as well as their sources of bias and variability. Any reported relaxation time is the result of the combination of the subject, hardware, acquisition, reconstruction algorithm, and map analysis that were used; consequently, all steps in obtaining a relaxation time can add bias or uncertainty to its measurement (Figure 1).

**Figure 2:**
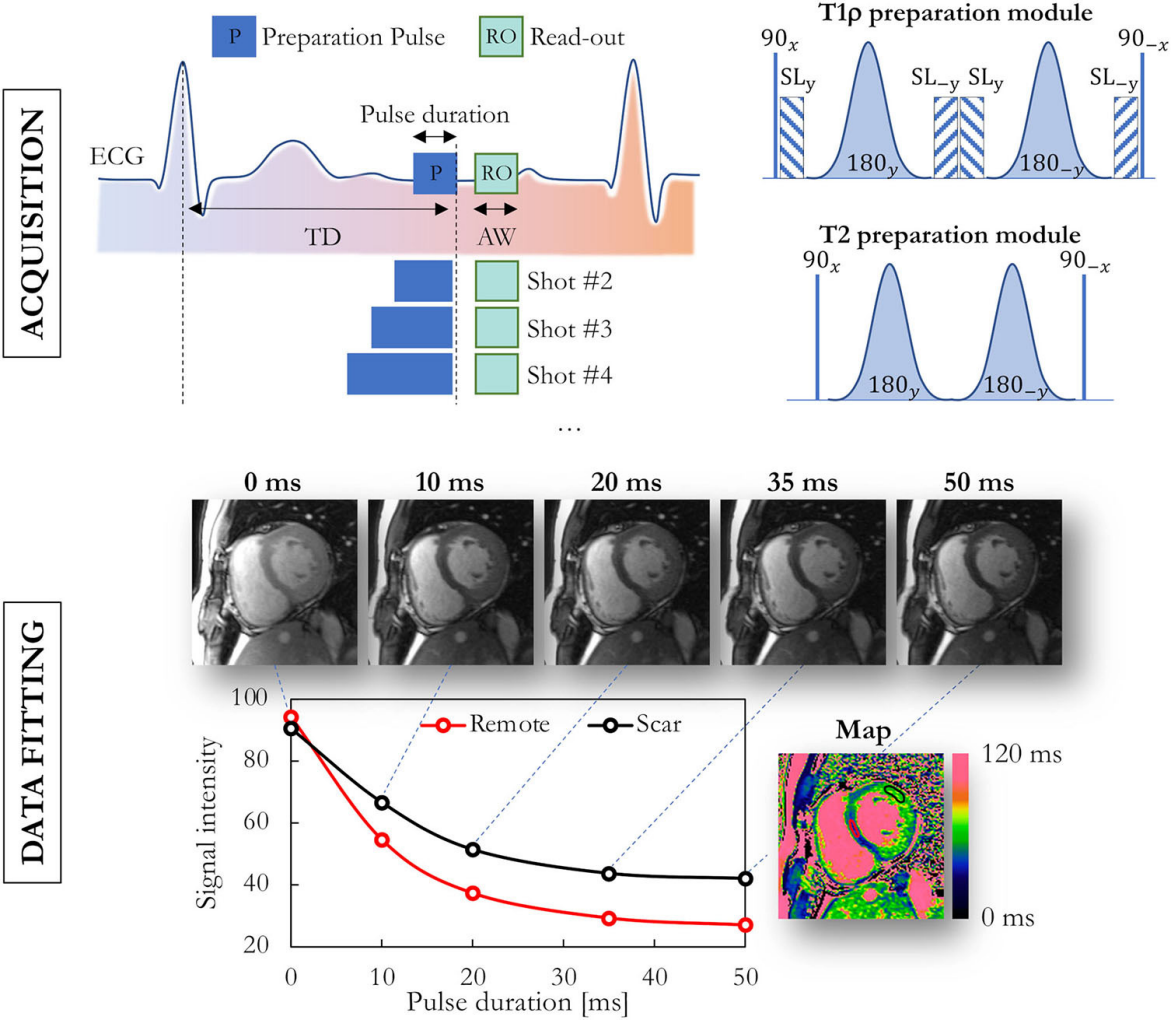
Data acquisition and fitting process in routine myocardial parametric mapping. Multiple images at the same location are acquired with different preparation modules, illustrated here for T_1ρ_ and T_2_ mapping. Semi-adiabatic T_1ρ_ and T_2_ preparation modules are shown on the top-right. The obtained images with varying contrast are then fitted on a per-pixel basis, and a false-color map is used to display the obtained values. TD, trigger delay; ECG, electrocardiogram; AW, acquisition window; SL, spin lock.

A relaxation time is commonly mapped by acquiring multiple images with a difference in the weighting time *t* of that relaxation time such that the dynamic range of the contrast is as large as possible (Figure 2). This enables the relaxation time to be fitted in each pixel, either with its own analytical equation as described above, or by matching the signal time course in the images to a premade dictionary (17).

### T_1_ Mapping

Many cardiac pulse sequences are currently used in the CMR community to directly quantify T_1_ values for each voxel in the myocardium. The earliest attempts to measure myocardial T_1_ values used the Look Locker inversion time (TI) scout method to characterized diseased tissue (18). Later on, Look Locker sequences were used to image the heart at different inversion times and estimate the final T_1_ map by fitting an exponential model through the corresponding pixels. While widely used, such a technique acquires the slices at different cardiac phases, and thus the cardiac shape can vary between the slices, resulting in inaccurate T_1_ maps.

Myocardial T_1_ mapping with the Modified Look-Locker (MOLLI) sequence was proposed in 2004 by Messroghli et al. (19) as a variant of Look Locker acquisitions. This sequence consists of a single-shot bSSFP image acquisition that is ECG-triggered at end-diastole, allowing for the precise reconstruction of a T_1_ map by merging multiple inversion-recovery (IR) experiments according to their inversion times. The standard MOLLI protocol provides precise T_1_ maps over a wide range of T_1_ values that cover the myocardial signal curve (e.g., 11 T_1_-weighted images are usually acquired over 17 heartbeats) and can be used in both pre- and post-contrast administration. The order of acquisitions and waiting periods of this sequence is indicated as 5(3)3, meaning that 5 images are acquired in the 5 heartbeats after the first inversion pulse, followed by a 3-heartbeat waiting period and a second inversion with another 3 acquired images in 3 heartbeats.

A major disadvantage of MOLLI is that the curve fitting yields an “apparent” T_1_ of the tissue, rather than the “true” T_1_. The apparent T_1_, also known as ${\text{T}}_{1}^{*}$, is a function of the true T_1_, heart rate, and other imaging parameters, such as the flip angle, views per segment, and TR, which all contribute to its bias. This bias is furthermore not constant: it increases at higher true T_1_ relaxation times. Finally, ${\text{T}}_{1}^{*}$ is also sensitive to non-ideal slice profiles and RF transmission field (B1) inhomogeneities. Consequently, when using Look-Locker IR methods, ${\text{T}}_{1}^{*}$ is always shorter than the true T_1_.

Alternative MOLLI techniques have been proposed to alleviate some of these disadvantages. The use of a minimum number of seconds instead of heartbeats as timing between the inversion pulses, i.e., 5s(3s)5s instead of 5(3)3, removes most of the bias at higher heart rates (20). A shortened version of the pulse sequence called ShMOLLI uses a 5(1)1(1)1 scheme that require shorter breath holds and is insensitive to the heartrate (21).

The slice-interleaved T_1_ (STONE) technique (22) is a free-breathing multi-slice T_1_ mapping technique and is both more accurate and precise than MOLLI. Here, a lung-liver navigator is used to enable a longer acquisition of multiple slices. The available time is exploited by cycling through acquisitions of the different slices in subsequent heartbeats, thus allowing the magnetization in each slice to relax for multiple heartbeats between readouts and avoiding the issues with the apparent T_1_ relaxation time. Interestingly, it has similar precision and repeatability at 1.5T when the acquisition is GRE and when it is bSSFP (23).

**Figure 3:**
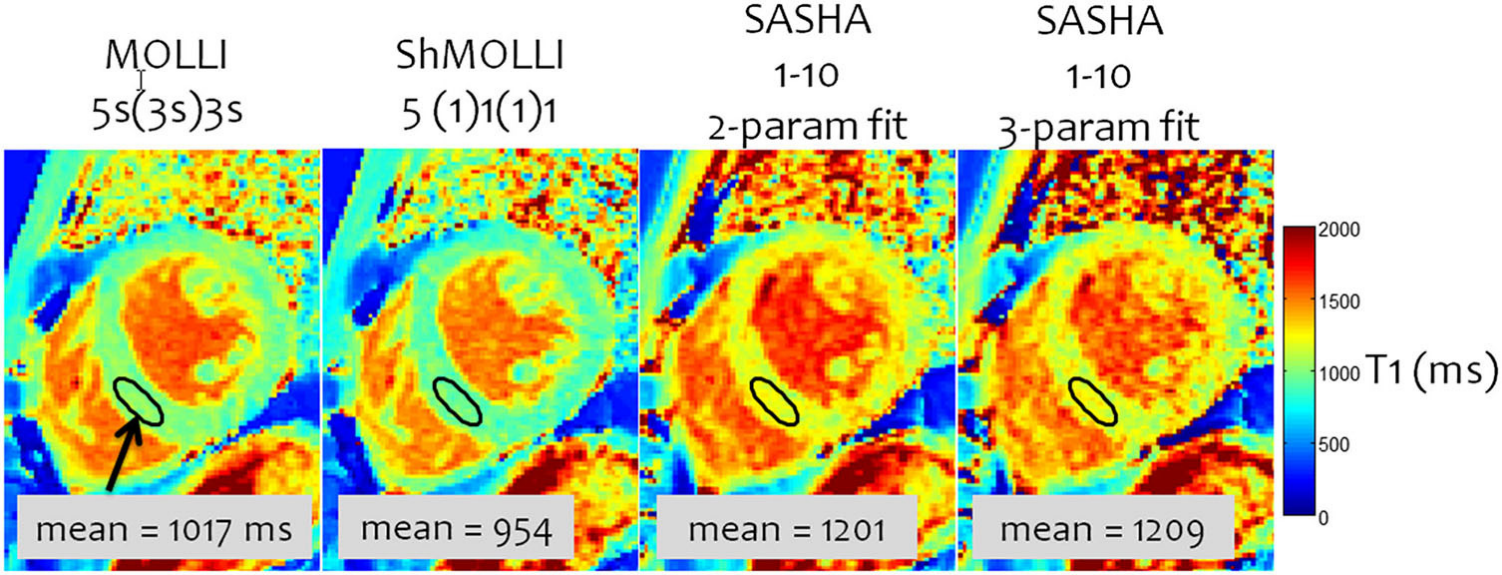
A comparison of inversion- and saturation-based myocardial T_1_ mapping sequences. MOLLI has the lowest variability due to the inversion and large number of samples, but the lowest accuracy because of the approximate nature of the Look-Locker correction and magnetization transfer. SASHA with a 2-parameter fitting has a small T_1_ underestimation; 3-parameter fitting is more accurate but has a significantly higher variability. Adapted from Kellman and Hansen (20) with permission.

The highest accuracy for T_1_ measurement is achieved with the single-point approach, where an image at a single delay time is acquired after each magnetization preparation. The magnetization preparation may be an inversion-recovery or a saturation-recovery (SR). Single-point imaging has the advantages that it is independent of most imaging parameters, insensitive to non-ideal slice profiles and B1 error, and directly measures the true T_1_, so no T_1_ correction methods are necessary. However, such techniques have a significantly lower precision than Look-Locker-based techniques due to both the limited amount of signal recovery that occurs within a heartbeat and the use of half the dynamic range. The SMART_1_Map pulse sequence (24) for example uses single-point SR bSSFP images and long magnetization recovery times after each saturation pulse by allowing multiple-heartbeat recovery times to accurately measure long T_1_ values. Saturation recovery single-shot acquisition (SASHA) similarly uses multiple shots (25), but uses an image without saturation preparation as an “infinite” recovery image (Figure 3). It also acquires 9 images with a shorter recovery time instead of 5 images with high SNR in order to improve precision.

The exact parameters that are used with the fitting model of Equation 1 affect the accuracy and precision of both inversion and saturation recovery based T_1_ mapping (20). For inversion, the recovery parameter A can be fixed at or very near 2 (due to T_1ρ_ recovery during adiabatic pulses), which then results in a two-parameter fit that maximizes precision at the cost of accuracy. Conversely, A can be left free in a three-parameter fit to account for field inhomogeneities and hardware imperfections, thus maximizing accuracy at the cost of precision due to the added degree of freedom. Similarly, for saturation recovery, A in Equation 1 can be fixed to 1 for a two-parameter fit and high precision, or it can be left free to improve accuracy (25).

While T_1_ mapping is normally performed in mid-diastole because of the relatively long rest period (and thus longer sampling time), it can also be performed at end-systole (26, 27). The end-systolic rest period is almost always more consistent than its mid-diastolic counterpart, and especially remains so during very high heartrates and episodes of frequent extrasystoles or atrial fibrillation (28, 29), when mid-diastolic imaging may become problematic. The T_1_ values themselves only show small and mostly non-significant differences between the two cardiac phases. If motion is correctly accounted for, the thicker myocardial wall may also contribute to lower partial-volume contaminations from neighboring blood and lipid tissue.

When a patient has an implanted cardiac device such as a pacemaker or implantable cardioverter-defibrillator (ICD), the B_0_ and B_1_ fields can be significantly distorted in the myocardium, and normal T_1_ mapping pulse sequences may result in large T_1_ estimation errors. The AIR (arrhythmia-insensitive rapid) T_1_ mapping pulse sequence avoids these sensitivities through several adaptations (30). These include GRE instead of bSSFP imaging and adiabatic saturation pulses to handle magnetic field inhomogeneities, as well as acquiring only two images to accelerate the acquisition. These adaptations result in a lower precision than IR- and bSSFP-based sequences such as MOLLI, although it has similar repeatability (31). A version of the AIR sequence that incorporates a wideband (8.9 kHz) saturation pulse was demonstrated to be even more robust in the presence of ICDs (32).

Saturation and inversion recovery preparations can also be combined to share the advantages of both, resulting in SAPPHIRE (33), which reduces the impact of high heart rates and arrhythmia. Here, each ECG trigger is directly followed by a SR pulse that removes all magnetization memory and thus the need for rest periods, insensitivity to heartrate variability, and increased short-T_1_ signal homogeneity. A subsequent IR pulse with a variable inversion time then adds a large dynamic range of T_1_ weighting. Like most mapping techniques that involve saturation, SAPPHIRE has a lower precision, higher accuracy and similar reproducibility as IR-based T_1_ mapping sequences (34).

There are several well-established clinical applications of T_1_ mapping. It has been shown to sensitively aid in the detection of fibrosis in myocarditis (35), amyloid deposition (36), iron overload (37), and Fabry's disease (38, 39). The combination of pre- and post-GBCA T_1_ maps can be used to calculate extracellular volume (ECV) maps, which are highly sensitive to diffuse fibrosis in conditions such as hypertrophic (40) and dilated (41) cardiomyopathy as well as chronic infarction (42) and cardiac allograft vasculopathy (43, 44). While native T_1_ mapping is sensitive to these diseases, it comes at the costs of limited specificity. Liu et al. found elevated T_1_ values in 13 out of 15 different tested cardiovascular diseases (45). One reason might be that mapping techniques sacrifice spatial resolution to yield quantitative information per pixel. This reduced spatial resolution of current mapping techniques is limiting its usefulness to detect typical intra-myocardial patterns of damage, e.g., to discriminate subendocardial from subepicardial damage, which is clinically highly relevant for diagnosis. Further developments of T_1_ mapping techniques might therefore aim at higher spatial resolution to allow for this intra-myocardial discrimination.

### T_2_ Mapping

Myocardial T_2_ mapping is primarily used for the diagnosis of (acute) edema and for the indirect detection of inflammation through such edema. Initially, breath-held turbo spin echo (TSE) pulse sequences were used (46, 47), but these were highly sensitive to motion due to the need for tissue to experience the entire refocusing pulse train. Interestingly, there has also been a minor comeback in TSE-based techniques in recent years (48), perhaps due to the availability of faster and more robust hardware.

**Figure 4:**
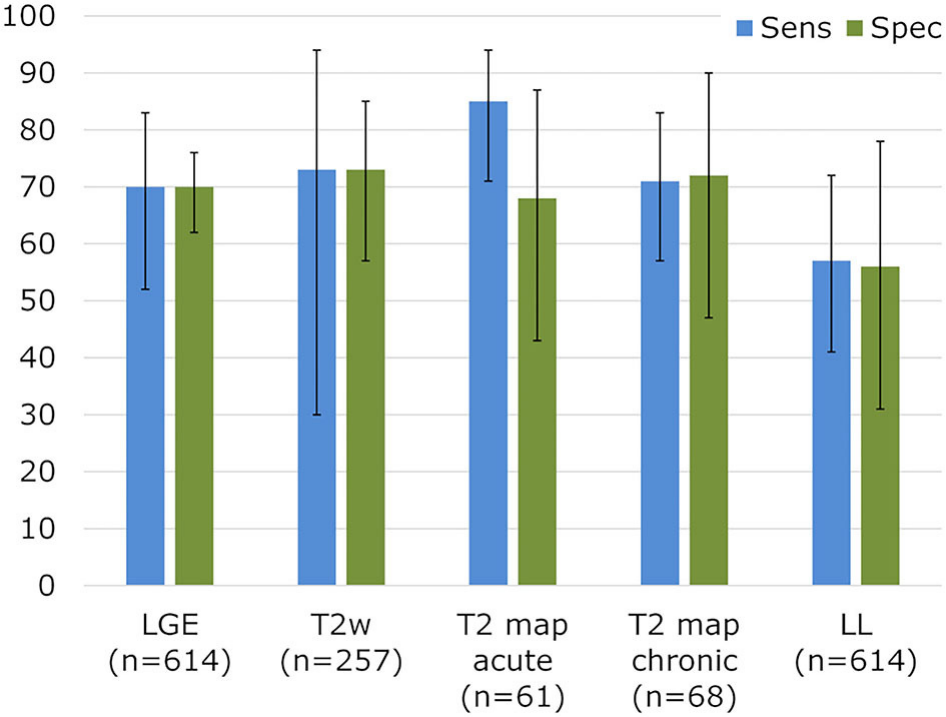
Sensitivity and specificity for established CMR parameters in biopsy-proven myocarditis. Diagnostic performance for myocarditis detection in a meta-analysis for LGE, T_2_-weighted imaging, and Lake Louise (LL) criteria. T_2_ mapping results vs. biopsies were collected from Lurz et al. (35). Adapted with permission from Mahrholdt et al. (53).

In recent years, TSE-based techniques have been mostly replaced by fast and motion-robust acquisitions that are preceded by a T_2_-preparation module (T_2_-prep). A T_2_-prep consists of an unlocalized set of RF pulses that tips down all magnetization, refocuses it as needed, and then tips it back up (Figure 2). This T_2_-prepared mapping was first described with spiral imaging (49) and then with Cartesian bSSFP (50) for BOLD MRI of the heart. These techniques were shortened to a single breath-hold acquisition by Giri and colleagues (51), which has become the most widespread myocardial T_2_ mapping technique. The T_2_ relaxation is characterized from 3 to 4 differently T_2_-weighted images that are each acquired within a single heartbeat (i.e., single-shot images). A relatively low number of robust and precise images are acquired in order to allow sufficient T_1_ recovery between these images and thus to mostly avoid a heartrate-dependent bias of the T_2_ fit, resulting in a technique that is robust and has low intra-observer, interobserver and inter-scan variability, and does not depend on heart rate (52). The technique has among others been shown to aid in the diagnosis of myocarditis (35, 53), where it had a high sensitivity compared to other CMR imaging techniques for the detection or exclusion of biopsy-proven myocarditis (Figure 4). T2 mapping has also been applied for the diagnosis of the area at risk after myocardial infarction (54, 55), the monitoring of the effects of chemotherapy on the heart (56), and acute rejection of the transplanted heart (57, 58).

Similar T_2_-prepared techniques were later optimized for free breathing with a navigator (59, 60), which allowed the technique to be applied in patient that struggle to hold their breath. T_2_-prep bSSFP was shown to also perform reasonably well at 3T (61), although more artifacts can be observed at higher magnetic field strength. Alternatively, gradient-recalled echo (GRE) imaging can be used to make the sequence more robust, although this comes at the cost of a sacrifice in precision (60, 62). A minor risk at low-SNR situations is that heavily T_2_-weighted signal may decay into the noise floor of the image, which may lead to artificially elevated T_2_ values. To avoid this, a fitting offset can be added (60, 63, 64). Similar to T_1_ fitting, leaving this offset free increases accuracy, while fixing it to a predetermined value increases its precision. T_2_ mapping can also be performed at end-systole in the case of very high heart rates or atrial fibrillation (26). While this forces a shorter acquisition window, it also leads to fewer partial volume effects. If faster mapping is desired, a saturation pulse can be added at the start of the acquisition, as always at the cost of precision (63). Several variations of the T_2_-prep module itself can be applied. The original version by Brittain and colleagues (65) can be set to be very short but is vulnerable to main magnetic field (B0) inhomogeneities, especially at higher magnetic field strengths. Alternatively, a semi-adiabatic version can be used (66), or a version with integrated fat-saturation capabilities (67, 68) to eliminate artifacts from bright lipids. These semi-adiabatic T_2_-prep modules use normal tip-down and tip-up pulses but have adiabatic refocusing pulses. It should be noted that the magnetization is spin-locked during these adiabatic pulses, and depending on their phase (69), T_1ρ_ or T_2ρ_ relaxation may occur instead of the desired T_2_ relaxation.

T_2_ gradient spin echo (T_2_-GraSE) (70–72) has more recently been adopted in the clinical setting as a robust and fast alternative to T_2_-prep bSSFP. GraSE imaging consists of a TSE sequence in which each of N echo is subdivided into a series of M echoplanar (EPI) readouts. The N = 6–9 TSE echoes are then used to generate a series of T_2_-weighted images to calculate the map, while the M = 3–7 EPI readouts per echo are used for spatial encoding and thus enable a faster acquisition. The technique has a similar performance in robustness, repeatability, and precision as T_2_-prep bSSFP and out-performs purely TSE-based T_2_ mapping (73). As its parent TSE, GraSE is still sensitive to motion artifacts, although to a lesser degree. Its EPI readout also requires well-calibrated gradient performance, which in turn requires a state-of-the-art scanner. GraSE has been successfully used for the diagnosis of hypertrophic cardiomyopathy (74), aortic stenosis (75), and myocarditis (76).

Several groups have explored the extension of T_2_-prepared cardiac T_2_ mapping to 3D in order to cover the entire heart and to detect small foci of inflammation. These techniques have been based on isotropic 3D radial bSSFP (77), Cartesian bSSFP (78), and stack-of-stars bSSFP (79). 3D T_2_ mapping has been used for detection of graft rejection (80), myocarditis (81), and inflammatory cardiomyopathy (82).

### ${\text{T}}_{2}^{*}$ Mapping

Myocardial ${\text{T}}_{2}^{*}$ mapping is the reference CMR technique for the diagnosis of diseases that involve an increase in myocardial iron content (83). While ${\text{T}}_{2}^{*}$ mapping was originally performed as a series of single-echo GRE images with increasing TE (84), it is nowadays mostly performed with multi-echo GRE (ME-GRE) sequences. In ME-GRE, 8 or 9 images with different TEs are acquired in a single breath-hold (85). A drawback of this approach is the sensitivity to magnetic susceptibility (especially near the cardiac veins) and to magnetic field inhomogeneities (in the lateral segments nearer to the lungs), low precision, and blood partial volume effects. Because of the vulnerabilities, ME-GRE ${\text{T}}_{2}^{*}$ maps are commonly only evaluated in the septal segments. It should be noted that this is only a minor detractor in the case of most iron storage diseases, since they are diffuse pathologies. A dark blood preparation Fitting of the ${\text{T}}_{2}^{*}$ decay with an offset and cropping the images that decay into the noise floor (86) have been proposed by He and colleagues to decrease bias, but like the extra degree of freedom in T_1_ and T_2_ fitting, risks decreasing precision. More recently, a respiratory-navigated ME-GRE variant (87) was proposed to overcome the precision issues by averaging several images for each echo time.

${\text{T}}_{2}^{*}$ mapping has been shown to correlate well with the cardiac iron concentration (88), and its application in β-thalassemia major has led to a paradigm shift in the management of the disease (89): by introducing ${\text{T}}_{2}^{*}$ mapping into clinical routine, the mortality of β-thalassemia patients decreased by 71% in a large UK registry (90). The transferability of the diagnostic quality of ME-GRE has been validated in a multi-center trial (91).

Several groups have also studied use of ${\text{T}}_{2}^{*}$ mapping for the quantification of intramyocardial hemorrhage (IMH) after the revascularization of myocardial infarction (92). A recent study by Chen et al. found that ${\text{T}}_{2}^{*}$ mapping has a very high sensitivity and specificity for the detection of IMH, but that the detected IMH volume with a generally accepted absolute threshold of 20 ms underestimates the volume detected with a subject-specific ${\text{T}}_{2}^{*}$ threshold (93).

### T_1ρ_ Mapping

Myocardial T_1_-rho (T_1ρ_) mapping has emerged as a promising CMR tool to quantify myocardial fibrosis without injection of contrast agent. T_1ρ_ mapping is performed by playing out a variable T_1ρ_ preparation module before a fast acquisition, similar to T_2_-prepared T_2_ mapping (94). The T_1ρ_ -prep module consists of tip-down, refocusing and tip-up RF pulses, interspersed with continuous low-power spin-locking pulses (Figure 2). T_1ρ_ mapping of the heart was initially explored in animal models, mostly to discriminate between infarct and healthy myocardium (95–99).

**Figure 5:**
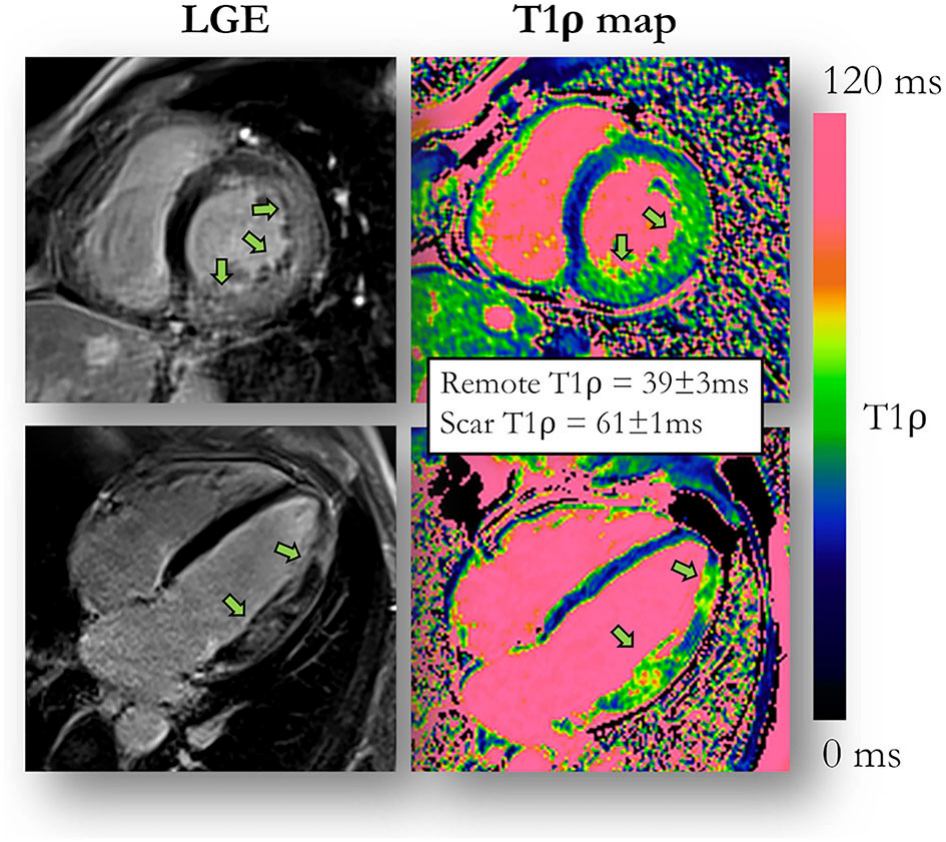
T_1_ρ mapping compared to late gadolinium enhancement (LGE) imaging. An example of a 51-year-old male patient with acute myocarditis and evidence of myocardial injury both on LGE images and contrast-agent-free myocardial T_1_ρ mapping. Adapted with permission from Bustin et al. (94).

Given the promise of gadolinium-free fibrosis quantification (Figure 5), T_1ρ_ mapping was initially explored in clinical studies of chronic infarction (100), hypertrophic cardiomyopathy (101, 102) and dilated cardiomyopathy (103). It was also successfully applied to map the myocardium in patients with end-stage renal disease where GBCAs could not be injected (104).

Most of these studies were applied with a spin-lock frequency around 500 Hz, which most likely leaves the T_1ρ_ values obtained from the different single-center studies as comparable to one another as those obtained with other mapping modalities. However, it is currently not clear to which degree the different T_1ρ_ preparation modules (with hard or adiabatic RF pulses, with and without phase cycling schemes) are comparable. For example, the adiabatic pulses are relatively long compared to part of the spin-lock durations, but spin-lock the magnetization at a different frequency.

## Recent Steps Toward Improved Reproducibility

The principal cause for the continued success of qualitative over quantitative CMR techniques can be found with the fast and straightforward encoding of MR data in qualitative imaging. In contrast to qualitative CMR, quantitative imaging tries to account for physical effects and interactions that happen during data collection to produce clinically valuable maps. This comes with a price: (i) lengthy acquisitions since multi-parametric information needs to be collected, (ii) inefficient data acquisition since most mapping techniques probe only one parameter at a time (e.g., T_1_ or T_2_), (iii) inaccurate maps since relaxation times are estimated using a simple exponential model that, by definition, is subject to some limitations, and (iv) a lower spatial resolution that hinders accurate and localized segmentation. Here we discuss recent technologies that have been designed to address the above obstacles.

### Acquisition and Reconstruction Strategies: Multiparametric Mapping and Beyond

**Figure 6:**
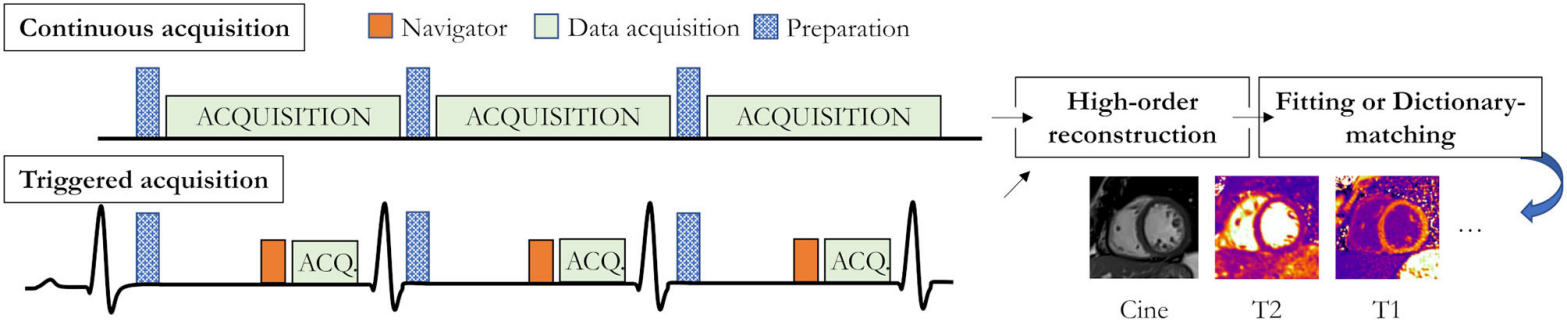
Recent acquisition strategies for single scan multiparametric cardiac mapping. Both accurate and precise continuous acquisition (top) and triggered acquisition (bottom) strategies have been proposed. Preparation modules could for example be an inversion pulse (as shown in the triggered diagram) or a T_2_ preparation module.

Recently described multiparametric mapping technologies take a step toward easier, faster, and more reproducible quantitative MRI of the beating heart by challenging the longstanding dominance of single-contrast-weighted imaging. These novel techniques promise to simplify myocardial mapping by for example providing simultaneous myocardial T_1_ and T_2_ maps and functional imaging with 2D or 3D coverage from a single scan. These technologies can be categorized into two groups: continuous and triggered acquisitions (Figure 6).

**Figure 7:**
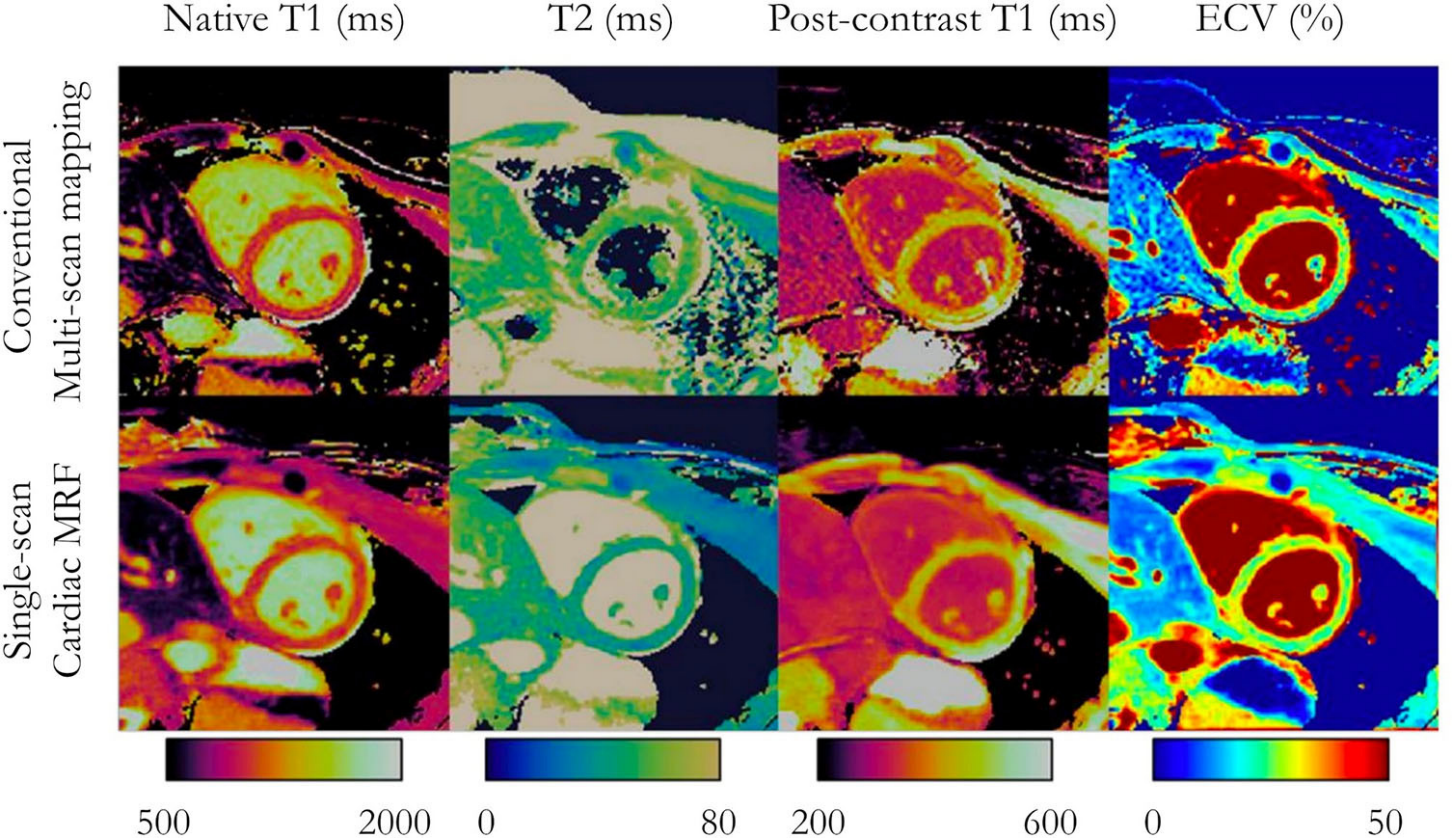
Multi-parameter mapping. Comparisons between conventional multi-scan native T_1_, T_2_, post-contrast T_1_, and synthetic ECV maps (top) with single-scan magnetic resonance fingerprinting (bottom) in a patient with no cardiac disease finding. Adapted with permission from Jaubert et al. (106). MRF, magnetic resonance fingerprinting; ECV, extracellular volume fraction.

*Continuous techniques*, such as MR fingerprinting (MRF) (17, 105, 106) or MR multitasking (107), attempt to capture the continuous transient state of the magnetization history with continuous data collection. By combining highly undersampled acquisition with variable modules (e.g., both saturation and inversion), and dictionary-based matching instead of the established curve fitting approaches, MRF offers co-registered multi-parametric maps with unprecedented speed. The cardiac MRF framework was initially proposed to simultaneously collect T_1_, T_2_, S_0_, and B_0_ maps with significantly faster scan times than conventional mapping techniques (Figure 7). It also promises several other advantages, such as the easy extension to other physical parameters (e.g., magnetization transfer, diffusion, ${\text{T}}_{2}^{*}$, and T_1ρ_), to biophysical model correction (e.g., integrating the slice profile, B_0_ field, or B_1_ field in the MRF dictionary), and to higher data encoding efficiency [e.g., simultaneous multi-slice (108) or 3D whole-heart].

Another uninterrupted MR technique for motion-resolved multiparametric cardiac mapping is the multitasking technique proposed by Christodoulou et al. (107). This technology aims to capture the multiple dynamics (e.g., cardiac and respiratory motion, parameter mapping, or contrast perfusion) in a unified ECG-free free-breathing framework. A high-order low-rank tensor decomposition framework is designed to naturally exploit the multiple dynamics given by this data-rich acquisition technology and to deliver high-quality cardiac maps in any given cardiac or respiratory states.

*Triggered techniques* acquire a small number of fully-sampled (or moderately undersampled) time-point images (often in mid-diastole) through the smart combination of preparation pulses (e.g., saturation, inversion, T_2_-preparation). Akçakaya et al. (109) proposed to interleave saturation-recovery and T_2_ preparation to acquire myocardial 2D T_1_ and T_2_ maps in a single breath-hold. Milotta et al. (110) took one step further to collect co-registered 3D whole-heart T_1_/T_2_ maps and water/fat imaging in a single 10-min free-breathing scan by combining inversion recovery and T_2_ preparation. These acquisition technologies promise to substantially reduce the scan duration, breathing instructions, and post-processing requirements, and thus increase parameter mapping reproducibility.

The application of artificial intelligence (AI) to improve map reconstruction is a very recent phenomenon. Nezafat and colleagues (111) have trained a neural network to remove streaking artifacts from radial T_1_ maps, while Guo et al. (112) trained a network to reconstruct precise T_1_ maps from only the first four images of a MOLLI pulse sequence, thus drastically shortening the needed breath hold. While these initial results are highly encouraging, it should be kept in mind that the single-center reproducibility of parametric mapping will likely also have its effect on the training of neural networks, suggesting that AI-enhanced mapping reconstructions may produce biases in other centers.

Finally, multi-parametric mapping is ideally suited to radiomics applications (113), where a large number of radiomic features is extracted from a single image (or map). Although, this goes beyond current clinical applications of parametric mapping, it may very well lead to significant knowledge discovery in a broad range of cardiac diseases. The first radiomic studies based on T_1_ mapping have shown to provide high diagnostic accuracy for the detection of microvascular obstruction (114) and hypertrophic cardiomyopathy phenotypes (115). However, it should be kept in mind that parametric mapping can only be included in radiomic analyses if the mapping techniques are consistent and reproducible in all patients; this may preclude its current use in multi-centric radiomic studies (116).

Unfortunately, the reproducibility of the above-mentioned technologies is still impacted by confounding factors. Continuous techniques are affected by slice profile and B_1_ imperfections (117–119) and by preparation pulse inefficiency (e.g., inversion, saturation, and T_2_-preparation). Mapping errors due to these imperfections can be significantly reduced by including these effects into the MRF dictionary (although this is computationally expensive), or by naturally acquiring data in 3D. Reproducibility can also be increased by considering intra voxel dephasing, off-resonance frequency, multi-compartment models, partial volume, and magnetization transfer variables (120–122) during dictionary generation.

### Post-processing Strategies: Fully Automated Quantification

Segmentation is an essential step to extract quantitative values from CMR parametric mapping. The delineation of regions of interest has remained a manual process in most clinical studies that use these parametric maps. Manual segmentation is a time-consuming process and is prone to subjective analysis errors inducing significant inter- and intra-observer variability. Furthermore, clinical recommendations do not necessarily suggest the segmentation of the entire heart, but only the delineation of some areas of pathology and healthy tissue for comparison (1). Several combinations of segmenting and reporting have been employed: (1) segmenting only a myocardial region with visibly elevated relaxation times together with a small apparently non-elevated region in the opposite myocardium (45, 51), (2) segmenting the myocardium according to the AHA guidelines (123) and reporting these (48, 73), (3) reporting a single whole-heart relaxation time (33, 43, 124), and (4) reporting single-slice relaxation times (36, 40). This lack of segmentation consensus is likely to affect reproducibility and comparison of parametric values between subjects and studies. Manual partial delineation of the myocardium might follow different guidelines depending on the cardiomyopathy (45). Given the possible inhomogeneity of parametric values in the heart, a complete segmentation of the myocardium, allowing division according to standard AHA segments, appears to be the most suitable approach going forward to provide reproducible results (73).

To enable more reproducible measurements of the values extracted from the parametric maps and to reduce the burden of manual analysis, automated segmentation methods are warranted. Automatic methods aim to provide a level of accuracy equal to the inter-expert variability of trained clinicians with extensive expertise in cardiac segmentation, and to reduce the duration of the segmentation. Such automatic methods would therefore remove the barrier for clinical use by non-experts by providing fast user-friendly tools that enable the consistent extraction of reproducible biomarkers from CMR parametric mapping. While several methods have been proposed for the segmentation of qualitative contrast-weighted imaging such as cine or late gadolinium enhancement images (125), only a few automated methods have so far addressed the issue of segmenting CMR parametric mapping to potentially offer a less operator-dependent process.

To correct the motion between the T_1_-weigthed images that are used for the generation of T_1_ maps, an active shape model approach has been proposed for the segmentation of the left ventricle blood and myocardium (126). This approach still required manual initialization and, as with all deformable models, it requires a substantial parameterization of the model, which is closely related to the nature of the datasets for which the model is trained. Consequently, it may be difficult to transfer this model to other types of CMR parametric mapping.

Deep learning (DL)-based approaches outperform traditional methods such as model-based and atlas-based methods, and have become the most promising solutions for CMR image segmentation. Using fully convolutional networks based on the U-Net architecture, methods have been proposed for the segmentation of native T_1_ maps of the left ventricular myocardium (127, 128) and together with the right ventricle (129). The integration of advanced features such as attention and densely connected layer mechanisms, has so far not yielded better results than a standard U-Net for myocardial segmentation of CMR parametric maps (128). However, since DL-based methods may be inconsistent for semantic medical image segmentation due to the high variability of the training dataset, quality control procedures can be incorporated to ensure the consistency of the computed segmentations. These quality controls can be additional modules that refine the segmentation via geometric *a priori* knowledge on the shape of the desired segmentations (127) or modules directly integrated to the neural network, which generates uncertainty information maps to reject inaccurate segmentation (129).

Although promising results have been reported for the segmentation on T_1_ native maps, one must keep in mind that the neural network training phase strongly relies on a tuning phase of the network hyperparameters that must be empirically performed, thereby reducing the fully automatic aspect of the method. Deep learning solutions must also be optimized by experts for a given dataset, and this commonly takes hours of implementation. The training models developed for one type of CMR image or map are therefore not directly applicable to other CMR modalities. Furthermore, DL-based methods derive part of their success from access to large databases. While there are several public databases of CMR cine or late gadolinium enhancement images (125), public databases of substantial annotated CMR parametric maps are still non-existent. These databases should also best represent the large phenotypic variability present in the disease states of the different cardiomyopathies where specific myocardial architectures must be considered. To face this issue of limited availability of manually segmented data, several methods have been proposed to artificially enlarge smaller datasets, such as data augmentation, transfer learning with fine-tuning, weakly and semi-supervised learning, self-supervised learning, and unsupervised learning. Leveraging transfer learning, Zhu et al. recently used a convolutional neural network pre-trained on T_1_ maps to automatically segment the left ventricular myocardium on T_2_ and ECV maps with an encouraging accuracy (130). Inter-modality registration methods also offer an alternative to modality domain change. Since DL-based methods perform better on qualitative images, Farrag et al. thus propagated a DL-based segmentation computed on cinematic images to T_1_ maps (128). However, this approach requires several specific acquisitions and faces the well-known inter-modality registration challenges.

## Outlook and Conclusion

Parametric mapping has become a routine part of CMR exams in clinical practice. These routine mapping techniques are often preferred over their qualitative counterparts due to multiple advantages, such as lower artifact ambiguity and facilitation of comparison between patients and throughout therapy. Furthermore, currently used techniques have an acceptable level of accuracy and precision, while their sources of bias and variability are well-understood. This renders these techniques appropriate for single-center studies and usage in routine exams. While full generalizability (131) may still be quite a way off, recent multiparametric and model-based map reconstructions enable the removal of many biases. For the adoption of parametric mapping by non-academic institutions, not only will inter-site reproducibility of the techniques need to be demonstrated, but the ease of use of the acquisition and analysis will also need to improve. Especially the reproducibility of acquisition planning and image segmentation remain significant rate-limiting steps. Free-running 2D techniques may improve the reproducibility of the scan planning, since fewer sequence timings need to be determined and set, while 3D free-running techniques may be less susceptible to anatomical confounders, since the entire heart is acquired in a standard anatomical orientation.

A sometimes-overlooked cornerstone of reproducibility is open access to sample data and source code: the sharing of datasets enables others to check their tools for bias against established techniques, while map reconstruction and analysis frameworks such as the Bay Area Reconstruction Toolbox (BART, https://mrirecon.github.io/bart/), the Michigan Image Reconstruction Toolbox (MIRT, https://github.com/JeffFessler/MIRT), and many others (4) can be used to harmonize map reconstruction. Efforts toward reproducibility and standardization can often be accelerated if there is an overarching international organization that many parties trust. To this end, several international networks such as the Quantitative Image Biomarker Alliance (QIBA) (132) of the Radiological Society of North America (RSNA) and the Quantitative MR Study Group (4) of the International Society for Magnetic Resonance in Medicine (ISMRM) have been established, and have put up roadmaps for the development of new quantitative imaging techniques that may help accelerate their acceptance for clinical practice.

From a clinical perspective, T_1_ mapping holds great promise to differentiate disease states from health. The quantification of ECV is of particular clinical interest for the diagnosis of cardiac amyloidosis, as some forms can now be treated successfully by novel drugs. Furthermore, the detection of Fabry's disease by low native T_1_ values is key for this diagnosis. On the other hand, most cardiac diseases are associated with elevated native T_1_ values, and without an etiological diagnosis, a targeted treatment is not possible. Therefore, clinical randomized controlled trials are needed to demonstrate the added value of native T_1_ measurements, since currently, an elevated T_1_ value does not lead to a direct management decisions. A specific technical aspect that may benefit from improvement relates to the spatial resolution of T_1_ mapping. In the past, a major goal of CMR imaging in post-infarct patients was the delineation of scar extent to decide for example on revascularization or on cardiac resynchronization therapy. Nowadays, the focus is on detection of arrhythmic substrates in patients at risk for ventricular tachycardias or sudden cardiac death to decide on ablation and placement of an implantable cardioverter defibrillator (ICD). This scar analysis requires very high spatial resolution, an aspect of T_1_ mapping that could be improved. The availability of T_2_ mapping techniques has improved the detection of myocardial edema due to fewer artifacts than typically associated with T_2_-weighted sequences. It also allows the quantitatively monitoring of disease activity and response to treatment, e.g., in rheumatic diseases, which affect the entire myocardium. Whether a T_2_-mapping-guided treatment approach in myocarditis or rheumatic disease is superior to a conventionally guided treatment needs to be documented by clinical trials. The application of ${\text{T}}_{2}^{*}$ mapping to guide therapy in thalassemia patients reduced mortality by 71% and is therefore the showcase model of how CMR can change patient outcome. Finally, T_1ρ_ mapping holds promise for the quantification of injury when GBCA cannot be injected, and remains to be further characterized and explored.

In conclusion, the advent of the mapping techniques could substantially improve our ability to accurately and reproducibly measure myocardial tissue characteristics. Current routine parametric mapping techniques have well-characterized sources of bias and variability and are widely accepted for single-center studies. In response to these challenges in reproducibility, a wide range of more accurate and precise techniques that leverage multiparametric and physical modeling, 3D coverage, deep learning, and automated segmentation have recently been developed. This increased reproducibility should be established though multi-center studies.

## Author Contributions

RvH, AB, and AO contributed to the conception and design of the manuscript. All authors contributed to manuscript revision, read, and approved the submitted version.

## Funding

This work was financially supported by the Swiss National Science Foundation (SNSF grants 32003B_182615 and CRSII5_202276 to RvH), by funding from the French National Research Agency under grant agreements Equipex MUSIC ANR-11-EQPX-0030, ANR-21-CE17-0034-01, and Programme d'Investissements d'Avenir ANR-10-IAHU04-LIRYC, and from the European Council under grant agreement ERC n715093 (to HC), and by Bayer Healthcare, Schweiz AG (to JS). AB acknowledges a Lefoulon-Delalande Foundation fellowship administered by the Institute of France.

## Conflict of Interest

JS declares research funding from Bayer Healthcare Schweiz AG. The funder was not in any way involved in this study. The remaining authors declare that the research was conducted in the absence of any commercial or financial relationships that could be construed as a potential conflict of interest.

## Publisher's Note

All claims expressed in this article are solely those of the authors and do not necessarily represent those of their affiliated organizations, or those of the publisher, the editors and the reviewers. Any product that may be evaluated in this article, or claim that may be made by its manufacturer, is not guaranteed or endorsed by the publisher.
